# Risk of reduced platelet counts in patients with nonalcoholic fatty liver disease (NAFLD): a prospective cohort study

**DOI:** 10.1186/s12944-018-0865-7

**Published:** 2018-09-19

**Authors:** Fang Liu, Hui Zhou, Lei Cao, Zhirong Guo, Chen Dong, Lugang Yu, Yiying Wang, Chunxing Liu, Jing Qiu, Yong Xue, Xingxiang Liu, Yunfang Xu

**Affiliations:** 1 Suzhou Center for Disease Control and Prevention, Suzhou, China; 2 Suzhou Industrial Park Centers for Disease Control and Prevention, Suzhou, China; 3 Xinghai Hospital of Suzhou Industrial Park, Suzhou, China; 4 Department of Epidemiology and Statistics, School of Public Health, Jiangsu Key Laboratory and Translational Medicine for Geriatric Disease, Medical College of Soochow University, Suzhou, Jiangsu China; 5 Huai’an Third Hospital, Huai’an, China; 6 Huai’an Forth Hospital, Huai’an, China

**Keywords:** Nonalcoholic fatty liver disease (NAFLD), Platelet counts, Cohort

## Abstract

**Background:**

The production of peripheral platelet is mainly regulated by thrombopoietin, which is a glycoprotein hormone predominantly synthesized in the liver. Previously, many studies have reported that there was an inverse correlation between the degree of chronic viral hepatitis and the peripheral platelet count. However, the effect of nonalcoholic fatty liver disease (NAFLD) on the peripheral platelet counts remains unclear.

**Methods:**

With 1303 participants from “The prevention of MS and multi-metabolic disorders in Jiangsu province of China (PMMJS)” cohort study, we investigated the associations between NAFLD and the risk of platelet counts reduction in Chinese adults. The paired-samples T test was used to explore the platelet counts changes between baseline and follow-up. Multivariate logistic regression was used to examine the association between presence of NAFLD and the risk of platelet reduction by calculating the odds ratios (ORs) and 95% confidence interval (CI).

**Results:**

After five years of follow-up, platelet counts were markedly reduced from 220.6 ± 42.22 (10^9^/L) at baseline to 208.41 ± 40.70 (10^9^/L) at follow-up in NAFLD group (*P* < 0.0001). However, platelet counts were slightly lowered from 213.2 ± 43.26(10^9^/L) at baseline to 211.8 ± 41.65 (10^9^/L) at follow-up in non-NAFLD people (*P* = 0.2349). Meanwhile, there was a significant association between NAFLD and the risks of platelet count reduction, even after adjustment for confounding variables (OR: 1.68, 95% CI: 1.06–2.67). Additionally, among the participants with BMI ≤ 23 kg/m^2^ and SUA ≤ 344.3 μmol/L, the NAFLD participants have an increased risk of platelet count reduction compared to the persons in non-NAFLD group.

**Conclusions:**

Our present results suggested that NAFLD individuals have an increased risk of platelet counts reduction.

**Electronic supplementary material:**

The online version of this article (10.1186/s12944-018-0865-7) contains supplementary material, which is available to authorized users.

## Background

Currently, nonalcoholic fatty liver disease (NAFLD) affected about 20–40% of population in Western countries, 5–40% of the general population across the Asia-Pacific region and 20% of adults in China [1, 2]. Therefore, NAFLD has become the most common chronic liver disease worldwide and is considered as one of the leading public health problems [3], although simple NAFLD is usually benign and the mechanism of the development and progression of the disease has not been fully understood.

The peripheral platelet production is mainly regulated by thrombopoietin, which is a glycoprotein hormone predominantly synthesized in the liver. Previously, a number of studies have reported that there was an inverse correlation between the degree of chronic hepatitis and the peripheral platelet count [4, 5]. Moreover, the platelet count itself and platelet-related index, such as AP index, APRI index and FIB4 index have been widely used to evaluate the severity of various liver diseases, especially in the patient with chronic hepatitis B or C virus infection [6, 7]. However, the effect of NAFLD on platelet count is debatable. The results from a Japanese study suggested that the platelet counts could be served as an ideal biomarker of the severity of fibrosis in NAFLD patients [8]. However, a more recent study in Iran reported that NAFLD has nothing to do with peripheral platelet counts [9].

In a community-based study, Dai et al. reported that platelet counts may start to fall earlier in course of HCV-induced liver diseases [10]. However, the study on the risk of platelet counts reduction at the early stage of NAFLD remains scarce. Considering the clinical syndrome of NAFLD is generally mild and most NAFLD cases do not progress to more advanced stages of liver disease [3, 11], we therefore performed the present study to analyze the prospective association between NAFLD and the reduced platelet counts based on 1303 Chinese adults from a PMMJS cohort study.

## Methods

### Study population

All of participants were recruited from the study of PMMJS, which was a prospective cohort study established at 2003–2004 and aimed to investigate the prevalence of metabolism syndrome in Jiangsu, China. The detailed design and protocol and sampling method of PMMJS have been described previously [12]. Briefly, total 5067 persons (aged 30–75 years) were selected by multi-stage sampling method and completed the standard questionnaire at baseline (February 2003-Augest 2003). Between February and August 2007, 4661 (406 lost) individuals were accepted the first follow-up and received the additional hepatic ultrasound examination for fatty liver disease diagnosis. In 2012, 4661 persons with hepatic ultrasound examination were invited to participate in additional follow-up survey and 4339 persons (322 lost) completed the follow-up examinations.

**Fig. 1:**
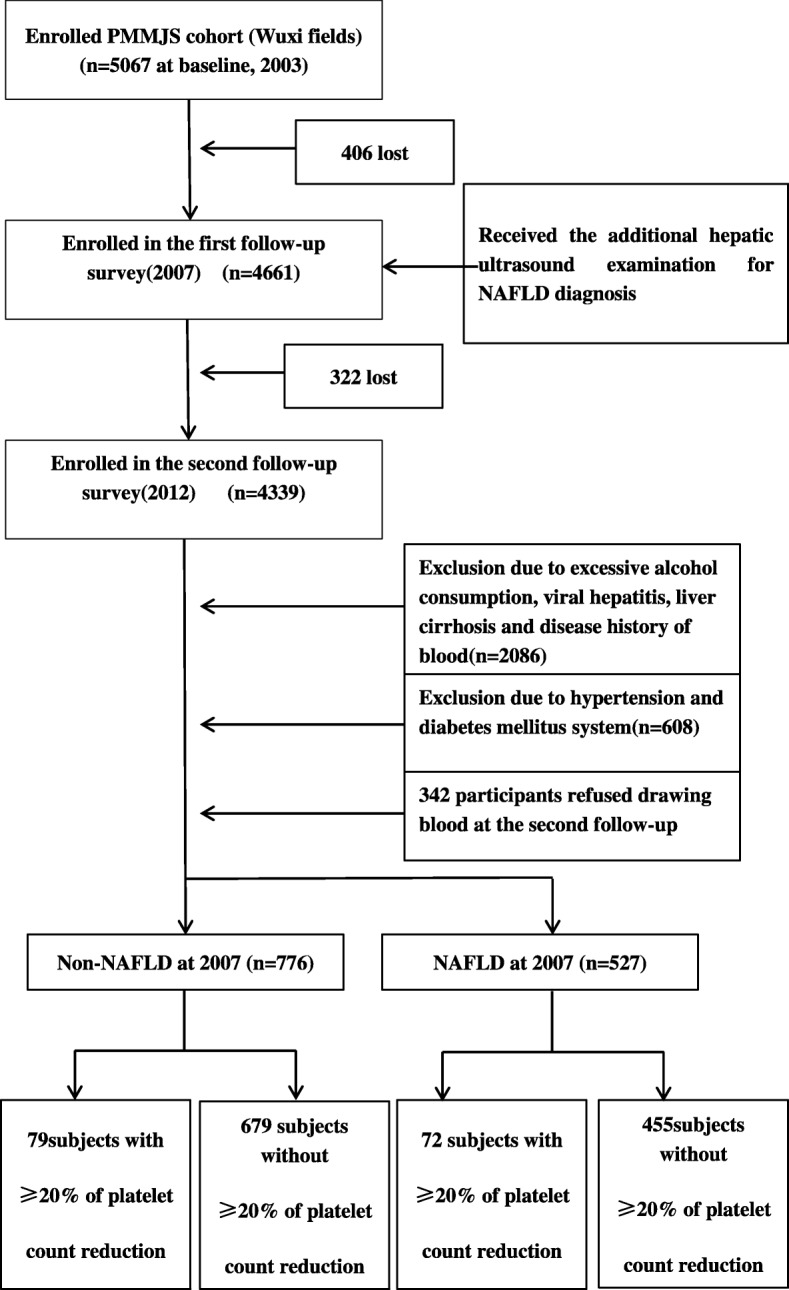
Study flow diagram for the included participant

In the present study, we excluded subjects with any of the following reasons: excessive alcohol consumption (≥ 30 g/day in men or ≥ 20 g/day in women) (1048), liver cirrhosis or suspicion of malignancy (39), schistosomiasis infection history (29), HBsAg positive (355) and anti-HCV antibody positive [10]. We also ruled out subjects with abnormal platelet count (93) or the disease history of blood system (512) including anaemia (504) and leukaemia [8] at baseline. Moreover, we excluded the participants with hypertension (312) and diabetes (296) in order to avoid the effects of anti-hypertension and anti-diabetes medicines on the peripheral platelet counts. Additionally, 342 elder persons (age > 65 years, mean age: 73.49 ± 7.27) who could not provide blood samples at the 2012 follow-up were also excluded. Thus, a total of 1303 subjects (aged: 30–60 years at baseline) were included for analysis (Fig. 1).

### Data collection and biomedical measurement

In the present study, the information collected in 2007 including the results of hepatic ultrasound examinations were used as baseline data. A standard questionnaire given to the participants contained the following: demographics (including age, gender, body weight, height and etc), smoking and drinking habits, disease history of hypertension and diabetes mellitus. Body mass index (BMI) was calculated by dividing weight in kilograms by height in meters squared. Blood pressure was measured three times using a mercury sphygmomanometer, with one minute interval between measurements after at least five minutes of rest in a seated position. The hepatic ultrasound examination was performed by an experienced radiologist, using a color ultrasound system (SoNoliNE Versa Plus, SIEMENS, German) with a 3.5-MHz probe.

A venous blood of 3 ml (ml) was collected in the morning after at least eight hours of fasting. Fasting plasma glucose (FPG), triglycerides (TG), total cholesterol (TC), LDL-cholesterol (LDL-C), HDL-cholesterol (HDL-C), serum uric-acid (SUA), serum creatinine (SCr), alanine aminotransferase (ALT), aspartate aminotransferase (AST) and γ-glutamyl transferase (GGT) were measured by using an autoanalyzer (Olympus AU640, Japan) with commercial reagents, respectively. The platelet count indices (PLT) were measured in EDTA-treated blood samples collected in 2–3 h, using an Advia 120 Hematology System (Bayer Corporation, Tarrytown, NY, USA) with commercial reagents.

### Definitions

NAFLD diagnosis was assessed according to “Chinese Guideline on Diagnosis and Treatment of NAFLD (2006)” [13]. It was determined based on the presence of at least 2 of the following 3 abnormal findings: diffuse increased echogenicity of liver relative to kidney, ultrasound beam attenuation, and poor visualization of intrahepatic structures. In addition, the alcohol consumption should be less than 30 g/day in males and 20 g/day in females, respectively.

According to the results from previous study [14], we defined the persons with the risk of platelet reduction if their platelet counts in the following-up examination (2012) decreased by 20% or more of their initial platelet count at baseline (2007).

### Statistical analysis

Data were analyzed using SAS 9.4 software (SAS Institute, Cary, NC, USA) and expressed as the mean ± standard deviation (SD), median or percentage as suitable. Comparisons between groups were analyzed by *t*-test or Mann–Whitney U-test for measurement data and Chi-square test for enumeration data. The incidence of platelet count reduction (decreased by more than 20%) was calculated for the NAFLD and non-NAFLD groups. Multivariate logistic regression was utilized to examine the association between presence of NAFLD and the risk of platelet count reduction by calculating the odds ratios (ORs) and 95% confidence interval (CI). In the multivariate models, some important confounders at baseline such as age, gender, smoking, BMI, systolic blood pressure (SBP), diastolic blood pressure (DBP), FPG, TG, ALT, AST, LDL-C and HDL-C level were included as covariates. All reported *P* values are two-tailed, and those < 0.05 were considered statistically significant.

## Results

### Baseline characteristics of studied population

Totally, 946 males and 357 females were included in the present study, with a mean age of 49.64 ± 8.15 years and 48.87 ± 7.65 years, respectively. Among them, 454/946 males were diagnosed as NAFLD, which was significantly higher than those in females (73/357, χ^2^ = 27.97, *P* < 0.001). As the results shown, the mean platelet count in the NAFLD and non-NAFLD subjects was 220.6 ± 42.22 and 213.2 ± 43.26 (10^9^/L) at baseline, respectively. There was a statistical difference between two groups (*P* = 0.0023). In addition, NAFLD and non-NAFLD subjects differed significantly in many parameters, such as BMI, ALT, AST, LDL-C, HDL-C and TG. Clinical and biochemical characteristics of study population were summarized in Table 1.

**Table 1:** Baseline characteristics of the study participants stratified by NAFLD

Variables	NAFLD	Non-NAFLD	*P* value
*N*	527	776	
Sex (%men)	454(86.15%)	492(63.40%)	*P* < 0.0001
Age (years)	52 ± 7.19	48 ± 8.42	*P* < 0.0001
BMI (kg/m^2^)	26.00 ± 2.57	22.49 ± 2.54	*P* < 0.0001
Current smokers (%)	212(40.23%)	216(27.84%)	*P* < 0.0001
SBP (mmHg)	119.5 ± 12.00	113.1 ± 12.31	*P* < 0.0001
DBP (mmHg)	79.00 ± 8.87	73.61 ± 8.69	*P* < 0.0001
Hypertension	142(26.94%)	84(10.82%)	*P* < 0.0001
Diabetes mellitus	45(8.54%)	17(2.19%)	*P* < 0.0001
ALT (U/L)	35.54 ± 22.92	20.52 ± 11.51	*P* < 0.0001
AST (U/L)	25.85 ± 10.19	20.69 ± 5.76	*P* < 0.0001
AST/ALT	0.85 ± 0.30	1.17 ± 0.41	*P* < 0.0001
Elevated ALT (> 40 U/L) (%)	154(29.22%)	53(6.83%)	*P* < 0.0001
GGT (U/L)	39.66 ± 37.51	23.05 ± 21.5	*P* < 0.0001
TG (mmol/L)	2.19 ± 1.65	1.31 ± 1.12	*P* < 0.0001
TC (mmol/L)	4.83 ± 0.82	4.61 ± 0.77	*P* < 0.0001
LDL-C (mmol/L)	3.00 ± 0.74	2.80 ± 0.69	*P* < 0.0001
HDL-C (mmol/L)	1.13 ± 0.26	1.36 ± 0.34	*P* < 0.0001
FBG (mmol/L)	5.73 ± 1.12	5.24 ± 0.71	*P* < 0.0001
SUA (umol/L)	381.8 ± 78.21	320.8 ± 77.45	*P* < 0.0001
SCr (umol/L)	74.86 ± 12.96	71.12 ± 13.56	*P* < 0.0001
PLT (10^9^/I)	220.6 ± 42.22	213.2 ± 43.26	0.0023

BMI: body mass index; SBP: systolic blood pressure; DBP: diastolic blood pressure; ALT: alanine aminotransferase; AST: aspartate aminotransferase; AST/ALT: aspartate aminotransferase/ alanine aminotransferase; Elevated ALT: Elevated Alanine aminotransferase; GGT: glutamyltransferase; TG: triglycerides; TC: total cholesterol; LDL-C: low density lipoprotein cholesterol; HDL-C: high density lipoprotein cholesterol; FBG: fasting blood glucose; SUA: serum uric-acid; SCr: serum creatinine; PLT: platelets

Numerical data were expressed as mean ± standard deviation or median. Categorical data were expressed as percentage

### Associations between NAFLD and the risk of platelet reduction

As the results shown, platelet counts were markedly reduced from 220.6 ± 42.22 (10^9^/L) at baseline to 208.41 ± 40.70 (10^9^/L) at the follow-up in NAFLD group (*P* < 0.0001). However, platelet counts were only slightly lowered from 213.2 ± 43.26 (10^9^/L) at baseline to 211.8 ± 41.65 (10^9^/L) at the follow-up in non-NAFLD group (*P* = 0.2349).

**Fig. 2:**
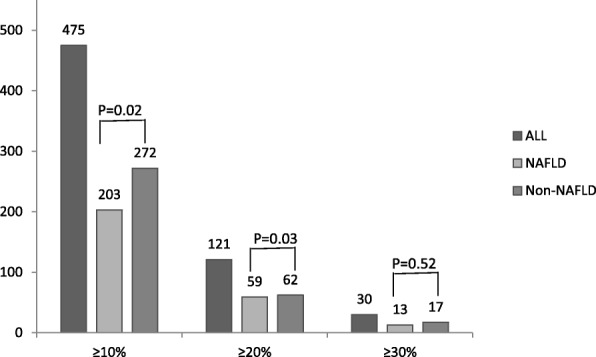
Age and sex-adjusted prevalence of ≥10%, ≥ 20% and ≥ 30% of reduced platelet counts of their initial counts at baseline in 1303 participants with and without NAFLD

As Fig. 2 shown, 203, 59, 13 NAFLD individuals and 272, 62, 17 non-NAFLD persons were determined with ≥10%, ≥ 20% and ≥ 30% of platelet counts reduction of their initial counts at baseline, respectively. When we defined the persons with the risk of platelet count reduction if their platelet counts in the following-up examination decreased by 20% or more of their initial count at baseline, 72 persons were determined with the risk of platelet reduction in NAFLD group, which was significantly higher than those in the non-NAFLD group (72/527 vs 79/776, *P* = 0.001).

In comparison with the individuals with non-NAFLD, NAFLD participants had an OR (95% CI) of 1.91 (1.31–2.78) for having an increased risk of platelet count reduction (≥20%) in unadjusted logistic models. As the results shown in Table 2, the significant association was not affected after adjustment for age, gender, BMI, FPG, SBP and DBP (model 1 and 2), and further adjustment for ALT and AST, SCr and SUA in model 3. Moreover, in fully adjusted models including HDL-C, LDL-C and TG, the results remained to show that the NAFLD significantly associated with the risk of platelet reduction (OR: 1.68, 95% CI: 1.06–2.67).

**Table 2:** Multivariate logistic regression analysis on the association between NAFLD and the risk of platelet count reduction^a^

	OR (95% CI)	*P* Value
Unadjusted association	1.91(1.31,2.78)	0.0007
Multiple adjusted association		
Model 1	1.77(1.14,2.72)	0.0113
Model 2	1.76(1.13,2.75)	0.0122
Model 3	1.93(1.22,3.04)	0.0047
Model 4	1.68(1.06,2.67)	0.0278

Data are express as odds ratios (95%CI) by univariate and multivariate logistic regression analysis. Participants *n* = 1477. Regression model adjusted as follows: Model 1: age, gender, BMI. Model 2: model 1 plus SBP, DBP, FPG. Model 3: model 2 plus AST, ALT, creatinine, uric-acid. Mode 4: Model 3 plus LDL-C, TG, HDL-C

^a^The platelet counts in the following-up examination (2012) decreased ≥20% of their initial platelet count at baseline (2007)

We further conducted stratified analyses to evaluate the effects of NAFLD on platelet count reduction. As the results shown in Table 3 and Additional file 1: Table S1, among the participants with BMI ≤ 23 kg/m^2^ and SUA ≤ 344.3 μmol/L, the NAFLD participants have an increased risk of platelet count reduction compared to the persons in non-NAFLD group (OR: 3.36, 95% CI: 1.20–9.45 for BMI, and OR: 3.69, 95% CI: 1.35–10.10 for SUA, respectively).

**Table 3:** Stratified analysis on the effects of NAFLD on the platelet count reduction in different subgroups

Variables				Unadjusted		Adjusted*	
		With outcome *N* (%)	Without outcome *N* (%)	OR (95% CI)	*P* value	OR (95% CI)	*P* value
Gender							
Male	Non-NAFLD	25(5.08%)	467(94.42%)	Ref(1)		Ref(1)	
	NAFLD	33(7.27%)	421(92.73%)	1.46(0.86,2.50)	0.1633	1.68(0.85,3.32)	0.1359
Female	Non-NAFLD	8(2.82%)	276(97.18%)	Ref(1)		Ref(1)	
	NAFLD	4(5.48%)	69(94.52)	2.00(0.59,6.84)	0.2689	3.47(0.53,22.97)	0.1943
Age (years)							
≤51	Non-NAFLD	16(3.52%)	438(96.48%)	Ref(1)		Ref(1)	
	NAFLD	9(4.21%)	205(95.79%)	1.20(0.52,2.77)	0.6654	1.46(0.47,4.54)	0.5188
> 51	Non-NAFLD	17(5.28%)	305(94.72%)	Ref(1)		Ref(1)	
	NAFLD	28(8.95%)	285(91.05%)	1.67(0.98,2.85)	0.0750	2.01(0.91,4.44)	0.0846
BMI (kg/m^2^)							
≤23	Non-NAFLD	19(4.14%)	440(95.86%)	Ref(1)		Ref(1)	
	NAFLD	8(15.09%)	45(84.91%)	4.12(1.71,9.94)	**0.0016**	3.36(1.20,9.45)	**0.0216**
> 23	Non-NAFLD	14(4.42%)	303(95.58%)	Ref(1)		Ref(1)	
	NAFLD	29(6.12%)	445(93.88%)	1.41(0.73,2.71)	0.3032	1.01(0.49,2.11)	0.9779
UA (umol/L)							
≤344.3	Non-NAFLD	17(3.53%)	464(96.47%)	Ref(1)		Ref(1)	
	NAFLD	14(8.19%)	157(91.81%)	2.43(1.17,5.05)	**0.0169**	3.69(1.35, 10.10)	**0.0100**
> 344.3	Non-NAFLD	16(5.42%)	279(94.58%)	Ref(1)		Ref(1)	
	NAFLD	23(6.46%)	333(93.54%)	1.20(0.62, 2.33)	0.5794	0.99(0.45, 2.21)	0.9844

BMI: body mass index; UA: uric-acid;

Values are OR (95% CI), participants *n* = 1303. Analysis according to gender distribution, median values of age, body mass index (BMI) and uric-acid (UA). When compared in different age groups, the OR was adjusted for gender, BMI, blood pressure and etc. When compared in different gender groups, the OR was adjusted for age, BMI, blood pressure and etc. When compared in different BMI groups, the OR was adjusted for age, gender, blood pressure and etc. When compared in different Uric acid groups, the OR was adjusted for age, gender, BMI, blood pressure and etc. *P*-values less than 0.05 were considered statistically significant

## Discussion

Although the platelet-related index has been used to predict degrees of fibrosis in the persons with chronic hepatitis [15], the characteristic of platelet counts variation at the early stage of NAFLD has not yet been illustrated by prospective, community-based study. Moreover, most NAFLD cases simultaneously suffer from hypertension or hyperlipidemia and routinely take antiplatelet medicines. Therefore, it is rational to perform the present study to evaluate the association between NAFLD and the risk of platelet count reduction in Chinese adults. As the results shown, our present study revealed that platelet counts were markedly decreased in NAFLD individuals compared to those people without NAFLD. Furthermore, among the participants with lower levels of BMI and SUA, the NAFLD participants have an increased risk of the platelet count reduction compared to those subjects in non-NAFLD group. The mechanism of NAFLD causing platelet counts reduction is multifactorial. Several studies have reported that lower platelet count was not only negatively correlated with liver fibrosis, but also associated with the progression of chronic viral hepatitis or alcoholic hepatitis [16–18]. Additionally, the liver is an important organ for thrombopoietin producing. During the development and progression of NAFLD, excessive lipid deposition and oxidative stress could impair the mitochondrial functions through inflammatory mediators and then affected thrombopoietin synthesis [19–21]. Reduced platelet counts could happen in this process.

In a Korean cross-sectional study, Shin et al. have observed that the increased values of mean platelet volume (MPV) was significantly associated with the prevalence of non-alcoholic hepatic steatosis in obese population, even after adjustment for confounding variables [22]. In a recent meta-analysis, Madan et al. also reported that MPV is significantly higher in patients with NAFLD, indicating the presence of increased platelet activity in such patients [23]. Therefore, the previous and present findings implicated that the characteristics of the peripheral platelet in NAFLD individuals should be further investigated in future. Furthermore, considering some drugs like aspirin could decrease the peripheral platelet counts and was generally used in the NAFLD patients co-existed with chronic metabolic diseases such as hypertension and hyperlipidemia [24, 25], more larger studies are needed to establish whether a platelet examination including platelet counts, MPV and etc., should be routinely performed in all NAFLD individuals.

Although the present longitudinal follow-up, prospective study considerably enabled us to further understand the risk of platelet reduction in NAFLD persons, some limitations in the present study should be noted. First, the impacts of portal pressure or splenomegaly on the platelet counts were not investigated in the present study. Second, the inflammatory immune changes in NAFLD individuals were not analyzed, which has been considered as an important factor associated with the platelet count decline in chronic hepatitis B or C patients [26, 27]. Third, we did not analyze the association between platelet count and NAFLD-related fibrosis/cirrhosis because fibrosis or cirrhosis should be determined by liver biopsy, and we did not collect the markers of NAFLD fibrosis/cirrhosis at baseline. This concern should be further explored in future study.

## Conclusions

Summary, our present study provided the evidence that NAFLD individuals have a significantly increased risk of platelet counts reduction compared to those without NAFLD. The results should be important for clinicians that the peripheral platelet counts need to be monitored in the NAFLD individuals, especially in the persons with antiplatelet medicines.

## Additional file

Additional file 1: **Table S1.** Stratified analysis of NAFLD and platelet count in different subgroup. (DOCX 27 kb)

## Abbreviations

ALT: Alanine aminotransferase
AST: Aspartate aminotransferase
BMI: body mass index
DBP: diastolic blood pressure
FBG: fasting blood glucose
GGT: glutamyltransferase
Hb: hemoglobin
Hct: hematocrit
HDL-C: high-density lipoprotein
LDL-C: low-density lipoprotein
NAFLD: nonalcoholic fatty liver disease
PLT: platelets
PMMJS: The prevention of MS and multi-metabolic disorders in Jiangsu province of China
SBP: systolic blood pressure
SCr: serum creatinine
SUA: serum uric-acid
TG: triglycerides
WBC: white blood cell count
