# Supplementary material for: Risk of reduced platelet counts in patients with nonalcoholic fatty liver disease (NAFLD): a prospective cohort study
Source: Lipids Health Dis. 2018 Sep 19;17:221. doi: 10.1186/s12944-018-0865-7 (PMC6145189; doi:10.1186/s12944-018-0865-7)
Supplement: Supplementary file 1 — Table S1. Stratified analysis of NAFLD and platelet count in different subgroup. (DOCX 27 kb) [file 12944_2018_865_MOESM1_ESM.docx]

Table S1 Stratified analysis of NAFLD and platelet count in different subgroup

| Variable |  | | |  | | Unadjusted | | Adjusted* | |
| --- | --- | --- | --- | --- | --- | --- | --- | --- | --- |
|  |  | | | With outcome | Without outcome | OR (95% CI) | *P* | OR (95% CI) | *P* |
|  |  | | | N (%) | N (%) |  |  |  |  |
| AST (U/L) | | | |  |  |  |  |  |  |
| ≤21 | Non-NAFLD | | | 21(4.17%) | 482(95.83%) | Ref(1) |  | Ref(1) |  |
|  | NAFLD | | | 14(6.90%) | 189(93.10%) | 1.70(0.85,3.41) | 0.1355 | 1.55(0.64,3.74) | 0.3347 |
| >21 | Non-NAFLD | | | 12(4.40%) | 261(95.60%) | Ref(1) |  | Ref(1) |  |
|  | NAFLD | | | 23(7.10%) | 301(92.90%) | 1.66(0.81,3.41) | 0.1652 | 1.94(0.77,4.93) | 0.1624 |
| ALT (U/L) | | | |  |  |  |  |  |  |
| ≤21 | | Non-NAFLD | | 19(3.69%) | 496(96.31%) | Ref(1) |  | Ref(1) |  |
|  | | NAFLD | | 12(8.33%) | 132(91.67%) | 2.37(1.12,5.01) | **0.0235** | 1.64(0.64,4.21) | 0.2994 |
| >21 | | Non-NAFLD | | 14(5.36%) | 247(94.64%) | Ref(1) |  | Ref(1) |  |
|  | | NAFLD | | 25(6.53%) | 358(93.47%) | 1.23(0.63,2.42) | 0.5440 | 1.76(0.76,4.06) | 0.1878 |
| SBP (mmHg) | | | |  |  |  |  |  |  |
| ≤140 | | Non-NAFLD | | 31(4.10%) | 726(95.90%) | Ref(1) |  | Ref(1) |  |
|  | | NAFLD | | 36(7.13%) | 469(92.87%) | 1.80(1.10,2.95) | **0.0200** | 1.78(0.94,3.38) | 0.0754 |
| >140 | | Non-NAFLD | | 2(10.53%) | 17(89.47%) | Ref(1) |  | Ref(1) |  |
|  | | NAFLD | | 1(4.55%) | 21(95.45%) | 0.41(0.03,4.85) | 0.4755 | >999.999 | 0.6086 |
| DBP (mmHg) | | | |  |  |  |  |  |  |
| ≤90 | | | Non-NAFLD | 32(4.25%) | 721(95.75%) | Ref(1) |  | Ref(1) |  |
|  | | | NAFLD | 31(6.39%) | 454(93.61%) | 1.54(0.93,2.56) | 0.0963 | 1.61(0.83,3.11) | 0.1573 |
| >90 | | | Non-NAFLD | 1(4.35%) | 22(95.65%) | Ref(1) |  | Ref(1) |  |
|  | | | NAFLD | 6(14.29%) | 36(85.71%) | 3.67(0.41,32.52) | 0.2433 | 6.69(0.49,91.97) | 0.1553 |
| Hypertension | | |  |  |  |  |  |  |  |
| Presence | | | Non-NAFLD | 4(7.02%) | 53(92.98%) | Ref(1) |  | Ref(1) |  |
|  | | | NAFLD | 10(11.24%) | 79 (88.76 %) | 1.68(0.50,5.63) | 0.4025 | 1.58(0.13,19.44) | 0.7230 |
| Absence | | | Non-NAFLD | 29(4.03%) | 690(95.97%) | Ref(1) |  | Ref(1) |  |
|  | | | NAFLD | 27(6.16%) | 411(93.84%) | 1.56(0.91,2.68) | 0.1038 | 1.78(0.88,3.60) | 0.1111 |
| SCr (umol/L) | | | |  |  |  |  |  |  |
| ≤73.7 | | | Non-NAFLD | 17(3.97%) | 411(96.03%) | Ref(1) |  | Ref(1) |  |
|  | | | NAFLD | 15(6.61%) | 212(93.39%) | 1.71(0.84,3.49) | 0.1405 | 1.51(0.55,4.16) | 0.4305 |
| >73.7 | | | Non-NAFLD | 16(4.60%) | 332(95.40%) | Ref(1) |  | Ref(1) |  |
|  | | | NAFLD | 22(7.33%) | 278(92.67%) | 1.64(0.85,3.19) | 0.1428 | 2.03(0.87,4.71) | 0.1004 |
| LDL-C (mmol/L) | | | |  |  |  |  |  |  |
| ≤2.88 | | Non-NAFLD | | 19(4.51%) | 402(95.49%) | Ref(1) |  | Ref(1) |  |
|  | | NAFLD | | 16(6.90%) | 216(93.10%) | 1.57(0.79,3.11) | 0.1988 | 1.28(0.49,3.33) | 0.6087 |
| >2.88 | | Non-NAFLD | | 14(3.94%) | 341(96.06%) | Ref(1) |  | Ref(1) |  |
|  | | NAFLD | | 21(7.12%) | 274(92.88%) | 1.87(0.93,3.74) | 0.0782 | 1.93(0.81,4.63) | 0.1383 |
| TC (mmol/L) | | | |  |  |  |  |  |  |
| ≤4.64 | | Non-NAFLD | | 18(4.30%) | 401(95.70%) | Ref(1) |  | Ref(1) |  |
|  | | NAFLD | | 16(6.84%) | 218(93.16%) | 1.64(0.82,3.27) | 0.1645 | 1.68(0.68,4.17) | 0.2606 |
| >4.64 | | Non-NAFLD | | 15(4.20%) | 342(95.80%) | Ref(1) |  | Ref(1) |  |
|  | | NAFLD | | 21(7.17%) | 272(92.83%) | 1.76(0.89,3.48) | 0.1039 | 1.92(0.77,4.82) | 0.1639 |
| TG (mmol/L) | | | |  |  |  |  |  |  |
| ≤1.28 | | | Non-NAFLD | 17(3.27%) | 503(96.73%) | Ref(1) |  | Ref(1) |  |
|  | | | NAFLD | 6(4.29%) | 134(95.71%) | 1.33(0.51,3.43) | 0.5613 | 2.61(0.82,8.29) | 0.1043 |
| >1.28 | | | Non-NAFLD | 16(6.25%) | 240(93.75%) | Ref(1) |  | Ref(1) |  |
|  | | | NAFLD | 31(8.01%) | 356(91.99%) | 1.31(0.70,2.44) | 0.4024 | 1.46(0.68,3.15) | 0.3346 |
| FBG (mmol/L) | | | |  |  |  |  |  |  |
| <7.0 | | | Non-NAFLD | 31(4.06%) | 732(95.04%) | Ref(1) |  | Ref(1) |  |
|  | | | NAFLD | 36(7.37%) | 453(92.64%) | 1.88(1.15,3.08) | **0.0126** | 1.86(0.98,3.54) | 0.0585 |
| ≥7.0 | | | Non-NAFLD | 2(15.38%) | 11(84.62%) | Ref(1) |  | Ref(1) |  |
|  | | | NAFLD | 1(2.63%) | 37(97.37%) | 0.15(0.01,1.80) | 0.1340 | — | 0.2613 |

SBP: systolic blood pressure; DBP: diastolic blood pressure; ALT: alanine aminotransferase; AST: aspartate aminotransferase; TG: triglycerides; TC: total cholesterol; LDL-C: low density lipoprotein cholesterol; FBG: fasting blood glucose; SCr: serum creatinine;

Values are OR (95% CI), participants n =1303. Analysis according to median values of AST, ALT, SBP, DBP, LDL, TC, TG SCr and presence/absence of the hypertension. When compared in different AST groups, the OR was adjusted for age gender BMI blood pressure etc. When compared in different ALT groups, the OR was adjusted for age gender BMI blood pressure etc. When compared in different blood pressure groups, the OR was adjusted for age gender BMI etc. When compared in different SCr groups, the OR was adjusted for age gender BMI blood pressure etc. When compared in different LDL groups, the OR was adjusted for age gender BMI blood pressure etc. When compared in different TG groups, the OR was adjusted for age gender BMI blood pressure etc. When compared in different TC groups, the OR was adjusted for age gender BMI blood pressure etc.
